# Exploring potential mortality reductions in 9 European countries by improving diet and lifestyle: A modelling approach

**DOI:** 10.1016/j.ijcard.2016.01.147

**Published:** 2016-03-15

**Authors:** M. O'Flaherty, P. Bandosz, J. Critchley, S. Capewell, M. Guzman-Castillo, T. Aspelund, K. Bennett, K. Kabir, L. Björck, J. Bruthans, J.W. Hotchkiss, J. Hughes, T. Laatikainen, L. Palmieri, T. Zdrojewski, on behalf of the Euroheart II Steering Group

**Affiliations:** a Department of Public Health, University of Liverpool, L69 3GB, UK, 2; b Population Health Research Institute, St Georges, University of London, UK; c Icelandic Heart Association, Iceland; d Department of Pharmacology & Therapeutics, Trinity Centre for Health Sciences, St James's Hospital, Dublin, Ireland; e Department of Epidemiology & Public Health, University College Cork, Cork, Ireland.; f Department of Molecular and Clinical Medicine, Sahlgrenska Academy, Gothenburg University, Sweden; g Institute of Health and Care Sciences, Sahlgrenska Academy, Gothenburg University, Sweden; h Center for Cardiovascular Prevention, Charles University in Prague, First Faculty of Medicine and Thomayer Hospital, Prague, Czech Republic; i School of Veterinary Medicine, University of Glasgow, UK; j UKCRC Centre of Excellence for Public Health, Queen’s University, Belfast, UK.; k Department of Chronic Disease Prevention, National Institute for Health and Welfare, Helsinki, Finland; l Institute of Public Health and Clinical Nutrition, University of Eastern Finland, Kuopio, Finland; m National Center of Epidemiology, Istituto Superiore di Sanità, Rome, Italy; n Medical University of Gdansk, Department of Hypertension and Diabetology, Poland

**Keywords:** Coronary heart disease, Mortality trends, Policy modelling, Prevention, Food policy, Smoking, Physical activity

## Abstract

**Background:**

Coronary heart disease (CHD) death rates have fallen across most of Europe in recent decades. However, substantial risk factor reductions have not been achieved across all Europe. Our aim was to quantify the potential impact of future policy scenarios on diet and lifestyle on CHD mortality in 9 European countries.

**Methods:**

We updated the previously validated IMPACT CHD models in 9 European countries and extended them to 2010–11 (the baseline year) to predict reductions in CHD mortality to 2020(ages 25–74 years). We compared three scenarios: conservative, intermediate and optimistic on smoking prevalence (absolute decreases of 5%, 10% and 15%); saturated fat intake (1%, 2% and 3% absolute decreases in % energy intake, replaced by unsaturated fats); salt (relative decreases of 10%, 20% and 30%), and physical inactivity (absolute decreases of 5%, 10% and 15%). Probabilistic sensitivity analyses were conducted.

**Results:**

Under the conservative, intermediate and optimistic scenarios, we estimated 10.8% (95% CI: 7.3–14.0), 20.7% (95% CI: 15.6–25.2) and 29.1% (95% CI: 22.6–35.0) fewer CHD deaths in 2020. For the optimistic scenario, 15% absolute reductions in smoking could decrease CHD deaths by 8.9%–11.6%, Salt intake relative reductions of 30% by approximately 5.9–8.9%; 3% reductions in saturated fat intake by 6.3–7.5%, and 15% absolute increases in physical activity by 3.7–5.3%.

**Conclusions:**

Modest and feasible policy-based reductions in cardiovascular risk factors (already been achieved in some other countries) could translate into substantial reductions in future CHD deaths across Europe. However, this would require the European Union to more effectively implement powerful evidence-based prevention policies.

## 1 Introduction

Cardiovascular disease (CVD), particularly coronary heart disease (CHD), continues to be a significant health problem in Europe and globally. Despite a sustained decline in premature CVD morbidity and mortality over two decades, this continuing burden will exert significant pressure on future healthcare systems, particularly given the sustained population ageing that most European countries are experiencing [1]. Prevention of CVD is therefore still a high priority, as recently stressed by the World Health Assembly [2].

Poor diet, tobacco, alcohol and physical inactivity are recognized as key drivers of the CVD epidemic [3], [4]. Furthermore structural, population wide policies can achieve substantial improvements resulting in large and rapid reductions in mortality [5] while also being cost-saving, making them attractive from a public health perspective [6], [7]. Some countries across the European region are therefore actively promoting their own nutritional, physical activity and tobacco control policies. However, the picture is mixed and many of the actions are limited, focusing on health promotion and education rather than the more powerful taxation and regulatory policy options [8]. Still, these latter strategies are perceived as politically more challenging to implement and their potential benefits are thus often overlooked.

The aim of this study was therefore to assess the number of deaths from CHD preventable by a range of population wide structural interventions. These aimed to modify the main lifestyle-related cardiovascular risk factors (smoking, salt and saturated fat intake and physical activity level) in nine European countries participating in the EuroHeart II project (Czech Republic, Finland, Iceland, Italy, Republic of Ireland, Northern Ireland, Poland, Scotland, Sweden).

## 2 Methods

To forecast the number of deaths potentially preventable in 2020 we adapted original IMPACT CHD Models in each country [9], [10], [11], [12], [13], [14], [15], [16], [17], [18], [19]. The data that the IMPACT model used to translate changes in risk factors (systolic blood pressure, total cholesterol, physical activity and smoking) into mortality reductions are detailed in the Technical Appendix (A2.1–2.4), and description of the general modelling methods is available in previous publications [20]. The original model estimates the deaths prevented or postponed (DPPs) that may be attributable to specific risk factor changes or treatment changes over a period of time. Here we summarize the methodology used to extend the IMPACT model to explore further policies around changes in specific nutrients (salt and saturated fat intakes) and perform forecasts of future mortality to 2020.

### 2.1 Estimating future trends in CHD mortality to 2020

In order to estimate future CHD mortality in 2020 for each country, an exponential decay model was fitted using Matlab. This took account of historical CHD mortality rates (annual trends by 10 year age group (from ages 25–74) and sex in each country. Annual trend data on CHD mortality was available over a period varying between 17 and 26 years for each country i.e. from the 1980s or 1990s up to the year 2010–11). Official population projections were obtained from national statistics agencies for the year 2020. This allowed us to capture both change in population structure and ongoing change in risk of mortality. This may therefore be a more realistic future mortality scenario compared to the traditional indirect standardization method that only captures population demographic changes, and simplistically assumes that CHD risk will remain constant at baseline levels. CHD mortality trends and population projections were obtained from national statistical offices in each country. For comparison, our analysis presented both the exponential decay model counterfactual (“continuing decline in mortality”) and the indirect standardized one (“no change in mortality”).

### 2.2 Updated IMPACT CHD model: translating changes in salt and saturated fat intake into mortality reductions

Using dichotomous risk factor values, the impact of future levels of physical inactivity and smoking on CHD mortality to 2020 was quantified using a population attributable change based method, as in previous work [20].

The original IMPACT model had no functionality to calculate deaths prevented or postponed (DPPs) according to changes in salt consumption and saturated/unsaturated fatty acids intake. For this project, we adapted the national IMPACT models by creating two additional layers to translate the effects of changes in these risk factors to changes in blood pressure and total cholesterol levels, as previously developed for analysis in the UK and Ireland [21], [22], [23]. Translating the effect of salt intake variation to changes in blood pressure was based on data published in a Cochrane systematic review [24] which quantified the effect of salt reduction on blood pressure in hypertensive and normotensive patients. We then used conventional IMPACT modelling methodology to translate the change in SBP levels into mortality reductions (see A2.1). In order to model the effect of saturated fat intake on serum cholesterol levels, we used the Clarke equations [25] to translate a change in saturated fat intake into a change in total cholesterol levels, assuming iso-caloric replacement with polyunsaturated and mono-saturated fats (assuming that each 1% absolute reduction in energy from saturated fat was replaced by 0.1% energy from mono- and 0.9% energy from poly-unsaturated fats). We estimated the effect of changes in multiple risk factors using a multiplicative approach, based on the methods used by Bajekal et al. [20].

**Fig. 1:**
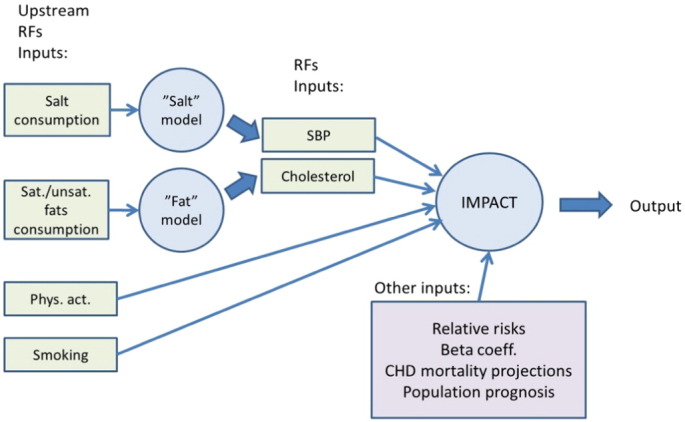
Structure of updated IMPACT CHD model.

The structure of the updated model is presented on Fig. 1.

### 2.3 Policy scenarios

Three policy scenarios were modelled to quantify the impact of potential future changes in risk factors on future CHD mortality. The first (S1), most conservative scenario assumed a small decrease in energy from saturated fats (and replacing it with energy from mono- and polyunsaturates), a modest 10% decrease in current salt intake, and a 5% decrease in the prevalence of smokers and physically inactive people.

The most optimistic scenario (S3) assumed improvements already achieved in exemplar countries. A salt intake reduction of 30% (as in Finland and Japan) [26], [27]; a 15% decrease in the prevalence of smoking (Australia and California) [28], [29] and 15% decline in physical in activity (Finland, Cuba) [30], [31]; and an absolute decrease of 5% in energy from saturated fats (Finland [32]).

Finally, the intermediate and plausible scenario (S2) used reductions between the conservative and optimistic ones, and its feasibility taking into account the baseline levels of the behavioural and biological risk factors are presented in the appending (Table A1.1 and A1.2, Technical Appendix).

Finally, we estimated the maximum theoretical reductions in CHD mortality to 2020 achievable in each country with “optimal” risk factor profiles for both smoking (assuming no-one in the country ever smoked) and physical inactivity (no inactive individuals).

### 2.4 Sensitivity analysis

We quantified the degree of stochastic uncertainty using Monte Carlo simulation implemented with R software. We repeated random draws from specified distributions for the input variables to iteratively recalculate the model. We calculated the uncertainty intervals based on 10,000 draws taking the 95% uncertainty intervals as the 2.5th and 97.5th percentiles. Input variables taken from external sources (e.g. beta coefficients and relative risk reductions) were randomly drawn from specified distributions. Distributions used for main input parameters are listed in the Technical Appendix (Table A3).

## 3 Results

In 2020, in all studied populations we expect approximately 63,800 (56,100–71,500) deaths assuming no changes in future mortality or just 37,900 (31,900–44,200) deaths if current trends continue to decline (Table 1, detailed mortality projections results are presented in Table A4).

For the most conservative scenario the forecast decrease in the expected number of deaths was 10.8% (7.3–14.0). Corresponding reductions for the intermediate and optimistic scenarios were 20.7% (15.6–25.2) and 29.1% (22.6–35.0) respectively. The forecast reductions in the number of deaths prevented or postponed (DPPs) varied between different countries (Table 2). For most optimistic scenario, relative decrease in deaths ranged from 27.2% (15.3–40.6) in Finland to 31.7% (16.5–48.9) for Sweden.

The absolute number of deaths prevented or postponed (DPPs) depended on the future mortality assumption. Assuming a continuation of current decreasing mortality trends, the absolute number of DPPs in all countries was estimated as approximately 4080 (2760–5290), 7830 (5900–9550) and 11,050 (8550–13,260) for the conservative, intermediate and optimistic scenarios respectively. If mortality did not decline, these forecast numbers of deaths prevented of postponed would be significantly higher: approximately 6900 (4820–8720), 13,240 (10,340–15,650) and 18,670 (14,990–21,800) respectively for the three scenarios. The absolute benefits differ between countries reflecting baseline mortality rates and population sizes (Table 2).

**Fig. 2:**
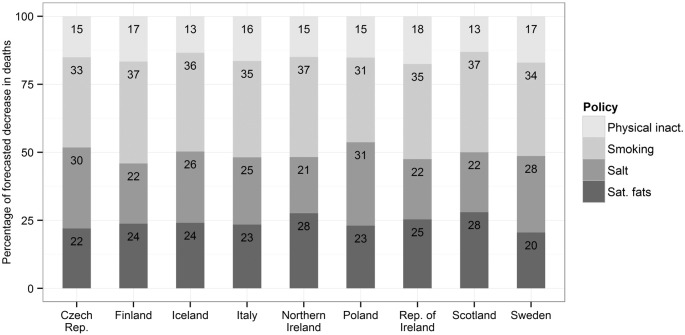
Percentage of total modelled CHD mortality reduction contributed by each policy (for “Conservative” scenario).

Nutrition-related policies resulting in reductions in salt and saturated fats and tobacco policies would deliver the largest gains. Together, they would account for approximately 80% of the reduction in expected future coronary heart mortality in these European countries (Fig. 2).

These gains from nutrition related policies occurred despite rather moderate reductions in modelled systolic blood pressure and total cholesterol values. The optimistic scenario for change in SBP across the different countries ranged from 1.9 to 3.0 mm Hg. The corresponding decrease in mean total cholesterol level was only 0.22 mmol/L.

The potential gains for the range of three policy scenarios were relatively consistent across the nine diverse European countries (see Fig. 2 and Appendix , Table A5.1–4). This is expected since the proportional reductions were the same in each country.

**Fig. 3:**
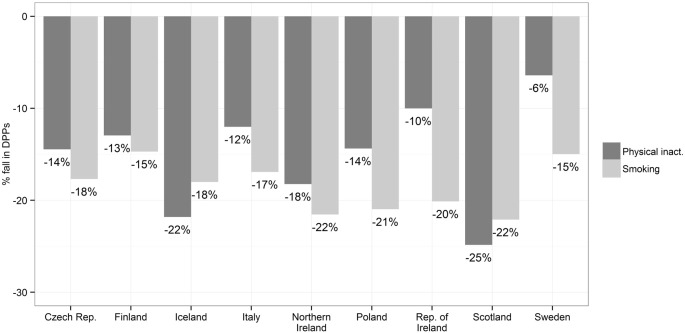
Reductions in mortality by country attributable to maximum theoretical improvements in physical inactivity and smoking (to achieve 0% prevalence).

However, Fig. 3 presents the maximum possible fall in DPPs from reductions in smoking and physical inactivity prevalence in each of the 9 countries. This demonstrated the amount of mortality fall theoretically possible if optimal levels of these risk factors could be achieved — idealistically assuming no-one smokes in the population and that there are no physically inactive individuals in any country. Countries where risk factor levels and mortality remain high (such as Scotland and Northern Ireland) have the greatest potential for future benefit from population wide policies to reduce these major risk factors.

## 4 Discussion

Feasible reductions in the main dietary and lifestyle risk factors in the nine studied EU countries could decrease future CHD mortality in 2020 and 2030 by approximately one third. The biggest gain would come from dietary improvements followed by decreases in smoking and physical inactivity. The results were reassuringly robust in the sensitivity analyses, and reasonably consistent across the 9 European populations included. These four lifestyle changes would therefore appear likely to meet the WHO NCD targets for a 25% relative reduction in the risk of premature mortality from CVD by 2025 from 2010 levels. The risk factor changes we modelled were similar to those recommended by the NCD targets; albeit slightly higher for reductions in physical inactivity and slightly lower for reductions in smoking. However, our analyses demonstrate the importance of changes in fat intake; which account for about one-quarter of the estimated reductions. This nutrient was not explicitly included as part of the WHO NCD targets; we recommend it should be included in the next revision of these targets and in all country targets for CHD prevention.

### 4.1 Comparison with other modelling studies

Our results are reassuringly consistent with results from other studies. A recent analysis in Ireland [22] predicted some 1070 fewer CHD and stroke deaths per year through healthier dietary intakes (specifically, increasing fruit and vegetable intake by 3 portions/day and reducing dietary salt by 3 g/day, trans-fats by 1% of total energy intake and saturated fats by 3% of total energy intake).

A previous UK study using a different modelling methodology suggested that feasible dietary improvements might reduce CVD deaths by approximately 20% [33], consistent with our estimates. This involved scenarios targeting a wider range of dietary risk factors (fruit and vegetables, fibre, total fat, saturated fat, monounsaturated fatty acids, polyunsaturated fatty acids, cholesterol and salt). The Global Burden of Disease study recently suggested that modest improvements in six dietary factors could achieve substantial reductions in the CVD burden by 2025 [34].

### 4.2 Strengths & limitations

Our study has several strengths. This modelling approach is comprehensive yet conceptually simple. It explores the potential impact of changes in major behavioural risk factors attributable to structural policies across a diverse range of European countries. It thus offers both regional and national level perspectives. We used the best available evidence from high quality nutritional survey data and systematic reviews of effect sizes to inform key model parameters. Estimating future mortality is highly uncertain, as shown by the rapid changes in mortality rates in Central Europe and Russia. Our model therefore uses a novel mortality counterfactual approach to explore a potential range of contrasting future baseline mortality scenarios, to help quantify where prevention could have the biggest impact on reducing future CHD mortality.

Our study also has some limitations. The modelling approach did not quantify competing risks for mortality. However, this effect was partly captured by the regression based mortality counterfactual, projecting current observed CHD mortality trends and population demographic projections. The risk factor effects were considered to be independent and additive. This might have overestimated the total reductions in mortality, despite our cumulative risk adjustment. Furthermore, the model included only CHD mortality outcomes. Reducing these risk factors would also have substantial benefits beyond coronary heart disease, by also decreasing stroke, common cancers, and cognitive decline [35], [36], suggesting that we have underestimated the impact of these changes on all cause mortality.

### 4.3 Public health implications

Our study demonstrates that moderate reductions in key life style behaviours would have a large impact on future CHD mortality and burden. However, recent trends in key risk factors across Europe have been less favourable than in earlier decades [37]. These changes in behaviours may thus not be achievable through education alone, but require fiscal and legislative responses i.e. further development of public health policy. This study forms part of the wider EU funded EuroHeart II Project (http://www.ehnheart.org/euroheart-ii.html). This has highlighted the fact that Central and Western European countries are at very different stages of developing public health policies to promote healthy diet and lifestyle strategies. For example, health education activities, dialogue, recommendations, guidelines, and patient information campaigns are widespread. They represent an early and uncontroversial part of the prevention policy process, but are considered to be only modestly effective. Conversely, taxation or regulation, are generally seen as more powerful strategies; but they remain uncommon, probably because they are considered politically more challenging [8], [38]. Specific regulations on salt, sugar and total fat are not widespread. Public health nutrition policies in Europe represent a complex, dynamic and rapidly changing environment. It is thus encouraging that the majority of European countries are engaged energetically in activities to improve their public health nutrition. However, most countries fall well short of optimal activities. Furthermore, our results highlight the potential opportunity costs of relying on less effective policies that rely on individual agency of healthcare providers and patients, like increasing the uptake of healthcare based interventions for primary prevention. Such downstream policies can achieve only small improvements in diet and smoking in the population as a whole, and risk widening inequalities [39]. Implementation of potentially powerful, “upstream” nutrition policies such as fiscal and regulatory interventions remains underused across Europe. A recent analysis from the Australian cost-effectiveness analysis programme (ACE) demonstrated that mandatory limits on salt in processed foods (particularly bread, margarine and cereals, resulting in reduction of 3–4 g a day of salt intake) would be the most cost-effective way of preventing premature CVD [40]. It also pointed out that current practise in the UK, Australian and elsewhere was inefficient, ignoring the most cost-effective policies and implementing policies (such as dietary advice or vascular health checks in primary care) which are not effective and have little population health benefits [40]. The scenarios modelled in our study also illustrate the potential benefits of structural policies with population wide effects, and in the case of smoking and physical activities, maximum possible gains by moving the entire population to a healthier lifestyle. Thus the biggest mortality gains could only be delivered if those risk factors were substantially reduced by regulation introducing or increasing taxation of salt and fat and tobacco [21], [41]. However, such policies sit higher up on the Nuffield ‘ladder of interventions’ [42]. Furthermore, the current direction of a major European wide policy initiative, the Transatlantic Trade Treaty, may pose a powerful threat to such policies [43]. The treaty negotiations are currently focused on protecting corporate interests while also undermining the power of states to regulate effective structural dietary and lifestyle policies [44]. The current EU diet and lifestyle policy environment is thus not particularly challenging or optimal at present, given that most of the current activity is not taxation or regulation but voluntary agreements with industry or individual level and educational interventions.

Our analyses highlight opportunities for massive health gains, and possibly still accruing after the time horizon in our analysis. However, this will need active policy engagement in identifying and implementing structural interventions. Although improvements in diet can rapidly translate into mortality reductions, these can also be quickly reversed [5], [31], [37]. Our modelled policy scenarios are politically feasible, and might be cautiously generalizable to other countries in Europe. They illustrate how successful policies in one country might translate into benefits when explored in a different setting. For example, government led reformulation policies in the UK, from 2003 to 2011, have resulted in salt reductions of about 1.5 g/day a (15% reduction, consistent with our optimistic scenario). Our modelling platform might therefore be potentially useful as a tool for prioritization and for supporting national and European debate on food policy.

### 4.4 Conclusions

Substantial reductions in coronary mortality could be gained by eminently feasible risk factor reductions achievable through population-wide diet and tobacco policies. These cost saving public health strategies should now be prioritized during the current debates shaping the future of healthy EU food policies.

## Competing interest

None declared.

## Figures and Tables

**Table 1:** Population and mortality projections (to 2020) for the EHII countries (adults 25–74 years).

Country	Population in base year [million]	Population in 2020 (forecast) [million]	Mortality in base year (per 100 000)	Mortality in 2020 (forecast) (per 100 000)	No of deaths observed in base year [thousands]	Expected no of deaths in 2020 (exponential decay model)	Expected no of deaths in 2020 (no mortality change)
Czech	6.8	7.3	145	67	9.9	4.9 (2.1–8.3)	10.6 (5.6–15.7)
Finland	3.3	3.5	113	58	3.8	2.2 (1.5–3.1)	4.4 (3.5–5.3)
Iceland	0.2	0.2	55	34	0.1	0.1 (0.1–0.1)	0.1 (0.1–0.1)
Ireland	2.7	3.6	74	52	2.0	1.5 (1.1–1.9)	2.1 (1.7–2.6)
Italy	38.6	38.0	40	27	15.6	11 (7.2–15.2)	16.2 (13–19.5)
Northern Ireland	1.1	1.2	83	39	0.9	0.4 (0.3–0.5)	0.9 (0.7–1.1)
Poland	25.1	25.6	91	56	22.8	13.6 (10.7–16.6)	22.2 (17.8–26.7)
Scotland	3.3	3.5	98	52	3.2	1.8 (1.2–2.4)	3.4 (2.7–4)
Sweden	5.8	6.3	59	37	3.4	2.4 (1.4–3.4)	3.8 (3.2–4.4)

**Table 2:** Total forecast prevented deaths according to three scenarios in study countries (all risk factors changes together). Adults aged 25–74 years old.

	Forecast decrease in deaths [n] (no mortality change)			Forecast decrease in deaths [n] (exponential decay model)			Forecast decrease in deaths [%]		
S1	S2	S3	S1	S2	S3	S1	S2	S3
Czech Republic	1160 (540–1860)	2240 (1060–3500)	3150 (1500–4880)	540 (210–950)	1030 (410–1790)	1450 (570–2520)	10.9 (4.2–19.5)	21.1 (8.3–36.7)	29.7 (11.7–51.5)
Finland	460 (280–640)	880 (570–1180)	1210 (810–1620)	230 (120–360)	440 (240–670)	610 (340–910)	10.3 (5.4–15.9)	19.7 (10.9–29.6)	27.2 (15.3–40.6)
Iceland	11 (7–14)	21 (15–26)	31 (22–38)	7 (4–9)	13 (9–17)	19 (13–25)	10.3 (6.8–13.7)	20 (13.8–26)	29.3 (20.2–38.2)
Italy	1740 (1110–2410)	3380 (2260–4500)	4710 (3220–6270)	1170 (660–1780)	2280 (1330–3340)	3170 (1890–4620)	10.6 (6–16.2)	20.7 (12.1–30.4)	28.8 (17.2–42)
Northern Ireland	90 (60–120)	170 (120–230)	250 (170–330)	40 (30–60)	80 (50–120)	120 (70–170)	10.3 (6.1–14.6)	20 (12.4–27.8)	29.1 (18.1–40.3)
Poland	2450 (700–4100)	4620 (2600–6640)	6530 (4150–8970)	1480 (430–2510)	2800 (1550–4060)	3970 (2480–5530)	10.9 (3.2–18.4)	20.6 (11.4–29.8)	29.2 (18.2–40.6)
Republic of Ireland	220 (140–310)	440 (290–580)	640 (430–840)	160 (90–230)	300 (190–430)	450 (280–620)	10.5 (6.3–15.2)	20.4 (12.9–28.7)	29.9 (18.7–41.7)
Scotland	330 (210–430)	640 (430–830)	930 (630–1220)	180 (100–260)	340 (200–490)	500 (300–720)	9.9 (5.7–14.3)	19.2 (11.4–27.6)	28.1 (16.6–40.3)
Sweden	440 (290–600)	860 (580–1140)	1220 (830–1600)	280 (140–440)	530 (280–840)	760 (390–1170)	11.5 (5.9–18.3)	22.4 (11.7–35)	31.7 (16.5–48.9)
Total	6900 (4820–8720)	13240 (10340–15650)	18670 (14990–21800)	4080 (2760–5290)	7830(5900–9550)	11050 (8550–13260)	10.8 (7.3–14)	20.7 (15.6–25.2)	29.1 (22.6–35)

S1 — conservative scenario, S2 — intermediate scenario, S3 — optimistic scenario.

All numbers of deaths rounded to nearest 10, except for Iceland.
