# Erratum to “Exploring potential mortality reductions in 9 European countries by improving diet and lifestyle: A modelling approach” [Int. J. Cardiol. 207 (2016) 286–291]

**DOI:** 10.1016/j.ijcard.2016.04.121

**Published:** 2016-07-01

**Authors:** M. O'Flaherty, P. Bandosz, J. Critchley, S. Capewell, M. Guzman-Castillo, T. Aspelund, K. Bennett, Z. Kabir, L. Björck, J. Bruthans, J.W. Hotchkiss, J. Hughes, T. Laatikainen, L. Palmieri, T. Zdrojewski, on behalf of the Euroheart II Steering Group

**Affiliations:** a Department of Public Health, University of Liverpool, L69 3GB, 2, UK; b Population Health Research Institute, St Georges, University of London, UK; c Icelandic Heart Association, Iceland; d Department of Pharmacology & Therapeutics, Trinity Centre for Health Sciences, St James's Hospital, Dublin, Ireland; e Department of Epidemiology & Public Health, University College Cork, Cork, Ireland; f Department of Molecular and Clinical Medicine, Sahlgrenska Academy, Gothenburg University, Sweden; g Institute of Health and Care Sciences, Sahlgrenska Academy, Gothenburg University, Sweden; h Center for Cardiovascular Prevention, Charles University in Prague, First Faculty of Medicine and Thomayer Hospital, Prague, Czech Republic; i School of Veterinary Medicine, University of Glasgow, UK; j UKCRC Centre of Excellence for Public Health, Queen's University, Belfast, UK; k Department of Chronic Disease Prevention, National Institute for Health and Welfare, Helsinki, Finland; l Institute of Public Health and Clinical Nutrition, University of Eastern Finland, Kuopio, Finland; m National Center of Epidemiology, Istituto Superiore di Sanità, Rome, Italy; n Medical University of Gdansk, Department of Hypertension and Diabetology, Poland

The publisher regrets publishing the author name incorrectly as K. Kabir. The correct name is Z. Kabir. The publisher would like to apologise for any inconvenience caused.

