# Supplementary material for: Exploring potential mortality reductions in 9 European countries by improving diet and lifestyle: A modelling approach
Source: Int J Cardiol. 2016 Mar 15;207:286–91. doi: 10.1016/j.ijcard.2016.01.147 (PMC4766942; doi:10.1016/j.ijcard.2016.01.147)
Supplement: Supplementary file 1 — Supplementary material. [file mmc1.docx]

# Technical Appendix

A1. Scenarios and baseline behavioral and biological risk factor levels.

Table A1.1 Three modelled scenarios parameter inputs: Conservative, Intermediate and Optimistic.

| Scenario | Absolute decrease in % energy from  saturated fats | Relative decrease decrease  in salt intake % | Absolute decrease in  prevalence of  physical inactivity  % | Absolute decrease of Smoking  prevalence  Prevalence  % |
| --- | --- | --- | --- | --- |
| Conservative (S1) | 1% | 10% | 5% | 5% |
| Intermediate (S2) | 2% | 20% | 10% | 10% |
| Optimistic (S3) | 3% | 30% | 15% | 15% |

**Table A1.2. Baseline Values of diet and lifestyle behaviors and associated biological risk factors, by country at the baseline year for the model (latest available year for each country)**

| **Country** | **SBP (mmHg)** | **Total Cholesterol (mmol/l)** | **Salt Intake (g/d)** | **Hypertension prevalence** | **Smoking prevalence** | **Physical Inactivity prevalence** | **Sat Fat Intake**  **(%E)** |
| --- | --- | --- | --- | --- | --- | --- | --- |
| Czech | 129.3 | 5.2 | 10.9 | 43.5% | 25.5% | 35.4% | 14.9 |
| Finland | 133.7 | 5.2 | 7.8 | 45.6% | 19.0% | 29.4% | 15.1 |
| Iceland | 121.5 | 5.1 | 8.1 | 29.5% | 21.5% | 49.6% | 14.5 |
| Ireland | 125.9 | 5.3 | 8.0 | 33.2% | 29.0% | 18.1% | 13.0 |
| Italy | 128.5 | 5.4 | 10.0 | 31.3% | 21.7% | 37.0% | 13.2 |
| Northern Ireland | 126.5 | 5.3 | 7.8 | 29.3% | 25.4% | 39.8% | 13.1 |
| Poland | 130.8 | 5.3 | 12.0 | 34.2% | 28.4% | 35.4% | 13.3 |
| Scotland | 129.4 | 5.3 | 8.8 | 28.3% | 24.8% | 63.9% | 12.3 |
| Sweden | 133.7 | 5.4 | 10.0 | 37.7% | 13.7% | 15.1% | 14.0 |

**A2. Risk Factors effect measures.**

Relative risk values and regression beta coefficients used in the IMPACT model used in this analysis are presented in tables A2.1-A2.4

Table A2.1 Beta coefficients for blood pressure change in population

| **Systolic blood pressure** | | **Age group (years)** | | | |  |
| --- | --- | --- | --- | --- | --- | --- |
|  | | **25-44** | **45-54** | **55-64** | **65-74** | |
|  | |  |  |  |  | |
| **Men** (hazard ratio per 20 mmHg) | | 0.49 | 0.49 | 0.52 | 0.58 | |
| Men (log hazard ratio per 1 mmHg) | | **-0.036** | **-0.035** | **-0.032** | **-0.027** | |
|  | |  |  |  |  | |
| *Minimum* | | *-0.029* | *-0.028* | *-0.026* | *-0.022* | |
| *Maximum* | | *-0.043* | *-0.042* | *-0.039* | *-0.032* | |
|  | |  |  |  |  | |
|  | |  |  |  |  | |
| **Women** (hazard ratio per 20 mmHg) | | 0.40 | 0.40 | 0.49 | 0.52 | |
| Women (log hazard ratio per 1 mmHg) | | **-0.046** | **-0.046** | **-0.035** | **-0.032** | |
| *Minimum* | | *-0.037* | *-0.037* | *-0.028* | *-0.026* | |
| *Maximum* | | *-0.055* | *-0.055* | *-0.042* | *-0.039* | |
|  | |  |  |  |  | |
|  | |  |  |  |  | |
| Source: Prospective studies collaborative meta-analysis, Lancet 2002 [1]. Minimum and maximum are ±20% of best estimate. | | | | | |  |
| Units: Percentage change in CHD mortality per 20 mmHg change in systolic blood pressure | | | | | |  |
| **Strengths:** | Large dataset, includes US data, adjusted for regression dilution bias, consistent with randomised controlled trials, results stratified by age and sex, with 95% confidence intervals | | | | |  |
| **Limitations:** | Some publication bias still possible | | | | |  |

Table A2.2 Beta coefficients for total cholesterol change in population.

| **Cholesterol** | **Age groups (years)** | | | | | | |
| --- | --- | --- | --- | --- | --- | --- | --- |
|  | **25-44** | | **45-54** | **55-64** | **65-74** | **75-84** | **85+** |
| **Mortality reduction (hazard ratio) per 1 mmol/l reduction in cholesterol** | | | | | | | |
| Men | 0.55 | | 0.53 | 0.36 | 0.21 | 0.21 | 0.21 |
| Women | 0.57 | | 0.52 | 0.35 | 0.23 | 0.23 | 0.23 |
| **Log coefficient (log hazard ratio)** | | | | | | | |
| **Men** | **-0.799** | | **-0.755** | **-0.446** | **-0.236** | **-0.117** | **-0.083** |
| *Minimum* | *-0.639* | | *-0.604* | *-0.357* | *-0.189* | *-0.093* | *-0.067* |
| *Maximum* | *-0.958* | | *-0.906* | *-0.536* | *-0.283* | *-0.140* | *-0.100* |
|  |  | |  |  |  |  |  |
| **Women** | **-0.844** | | **-0.734** | **-0.431** | **-0.261** | **-0.174** | **-0.051** |
| *Minimum* | *-0.675* | | *-0.587* | *-0.345* | *-0.209* | *-0.139* | *-0.041* |
| *Maximum* | *-1.013* | | *-0.881* | *-0.517* | *-0.314* | *-0.209* | *-0.062* |
| Source: Prospective studies collaborative meta-analysis, Lancet 2007 [2]. Minimum and maximum are ±20% of best estimate. | | | | | | | |
| Units: | | Percentage change in CHD mortality per 1 mmol/l change in total cholesterol | | | | | |
| **Strengths:** | | Includes US data, adjusted for regression dilution bias, includes randomised controlled trials, RCT values consistent with observational data, results stratified by age and sex, with 95% confidence intervals | | | | | |
| **Limitations:** | | Some publication bias still possible | | | | | |

Table A2.3 Relative risk and beta coefficients for mortality from Coronary Heart Disease for current smokers relative to non-smokers (95% CIs in parentheses), from the American Cancer Society’s Cancer Prevention Study (CPS-II)[3]

| **Age** | **Male** | **Female** |
| --- | --- | --- |
| **Relative risk** |  |  |
| **30-44** | 5.51 (2.47-12.25) | 2.26 (0.83-6.14) |
| **45-59** | 3.04 (2.66-3.48) | 3.78 (3.10-4.62) |
| **60-69** | 1.88 (1.70-2.08) | 2.53 (2.22-2.87) |
| **70-79** | 1.44 (1.27-1.63) | 1.68 (1.46-1.93) |
| **>=80 years** | 1.05 (0.78-1.43) | 1.38 (1.08-1.77) |
| **Beta coefficients (log RR)** | | |
| **30-44** | 1.71 (0.90-2.51) | 0.82 (-0.19-1.81) |
| **45-59** | 1.11 (0.98-1.25) | 1.33 (1.13-1.53) |
| **60-69** | 0.63 (0.53-0.73) | 0.93 (0.80-1.05) |
| **70-79** | 0.36 (0.24-0.49) | 0.52 (0.38-0.66) |
| **>=80 years** | 0.05 (-0.25-0.36) | 0.32 (0.08-0.57) |
| Notes: CPS-II is an ongoing prospective study of mortality in 1.2 million Americans aged 30 years or more when they completed a questionnaire on tobacco and alcohol use, diet, and multiple other factors affecting health and mortality in 1982. RRs were estimated from Cox proportional-hazard models, with non-smokers as the reference group (RR=1.0 for non-smokers). Risks were adjusted for age, race, education, marital status, "blue collar" employment in most recent or current job, weekly consumption of vegetables and citrus fruit, vitamin (A, C, and E) use, alcohol use, aspirin use, body mass index, exercise, dietary fat consumption and for hypertension and diabetes (both at baseline). Analyses of the hazards associated with smoking were based on the first six years of follow-up (1982 through 1988). Source: Ezzati et al (2005) [[6]](#_bookmark76) | | |

Table A2.4 : Relative risk and beta coefficients for Coronary Heart Disease from physical (in)activity levels from WHO GBD Study (95% CIs in parentheses), relative to those considered physically active[4]

| **Age** | **Inactive level** | | **Insufficiently active level** | | |
| --- | --- | --- | --- | --- | --- |
| **Relative risk** | |  | |  |  |
| **15-69** | | 1.71 (1.58-1.85) | | 1.44 (1.28-1.62) |  |
| **70-79** | | 1.50 (1.38-1.61) | | 1.31 (1.17-1.48) |  |
| **80+ years** | | 1.30 (1.21-1.41) | | 1.20 (1.07-1.35) |  |
| **Beta coefficients (log RR)** | |  | |  |  |
| **15-69** | | 0.54 (0.46-0.62) | | 0.36 (0.25-0.48) |  |
| **70-79** | | 0.41 (0.32-0.48) | | 0.27 (0.16-0.39) |  |
| **80+ years** | | 0.26 (0.19-0.34) | | 0.18 (0.07-0.30) |  |
|  |  | |  | | |
| Notes: Physical (in)activity in the WHO GBD study was treated as a categorical variable with three categories: Level  1: Inactive: doing no or very little physical activity at work, at home, for transport, or during discretionary time. Level 2: Insufficiently active: doing some physical activity but less than 150 minutes of moderate-intensity physical activity or 60 minutes of vigorous-intensity physical activity a week accumulated across work, home, transport or discretionary domains. Level 3: Sufficiently active (unexposed): at least 150 minutes of moderate-intensity physical activity or 60 minutes of vigorous-intensity physical activity a week accumulated across work, home, transport or discretionary domains, which approximately corresponds to current recommendations in many countries. RR estimates were adjusted for confounding variables, measurement error associated with self-report, and attenuated over age (25% of the excess risk for the 70-79 year age-group and 50% of the excess risk for the oldest age group, 80+), but not adjusted for blood pressure and cholesterol.  Sources: Bull et al (2004) [[4];](#_bookmark77) Joubert et al (2007) [[5]](#_bookmark78) | | | | | |

## A3. Sensitivity analysis parameters

Table A3.1 Distributions used for main input parameters in the model.

| Group | Parameters | Distribution | Distribution parameters |
| --- | --- | --- | --- |
| Population counts in base year and CHD deaths stratified by age and sex | Population counts (no error) | No error (uniform distribution) |  |
|  | CHD mortality (no error) | No error (uniform distribution) |  |
| Population counts in final year stratified by age and sex | Population counts | Normal(mean, SD) | Mean =point estimateSD=standard error of the mean |
|  | CHD mortality | Normal(mean, SD) | Mean =point estimateSD=standard error of the mean |
| Prevalence/mean estimates | Prevalence estimates (smoking physical activity, hypertension prevalence) – beta distribution. | Beta (alpha, beta) | Alpha=casesBeta= non-cases |
|  | Continuous variables (SBP, total cholesterol, salt intake) | Normal(mean, SD) | Mean =point estimateSD=standard error of the mean |
| Relative risk reduction | Relative risk for CHD deaths forsmoking and physical incativity | RelRisk(RR, SE ln(RR)) | RR=relative riskSE ln(RR) =standard error |
| Beta coefficients | Beata coefficients for quantifying relation of SBP and cholesterol level with CHD mortality | Normal (mean, SD) | Mean =point estimateSD=standard error of the mean |

## A4. Results of baseline mortality projections using exponential decay model.

Fig A4.1 Results of baseline projections of future mortality using exponential decay model. Red bars represent observed mortality in base year (in sex and age groups). Blue bars represent expected mortality in 2020 (with 95% confidence limits).

##
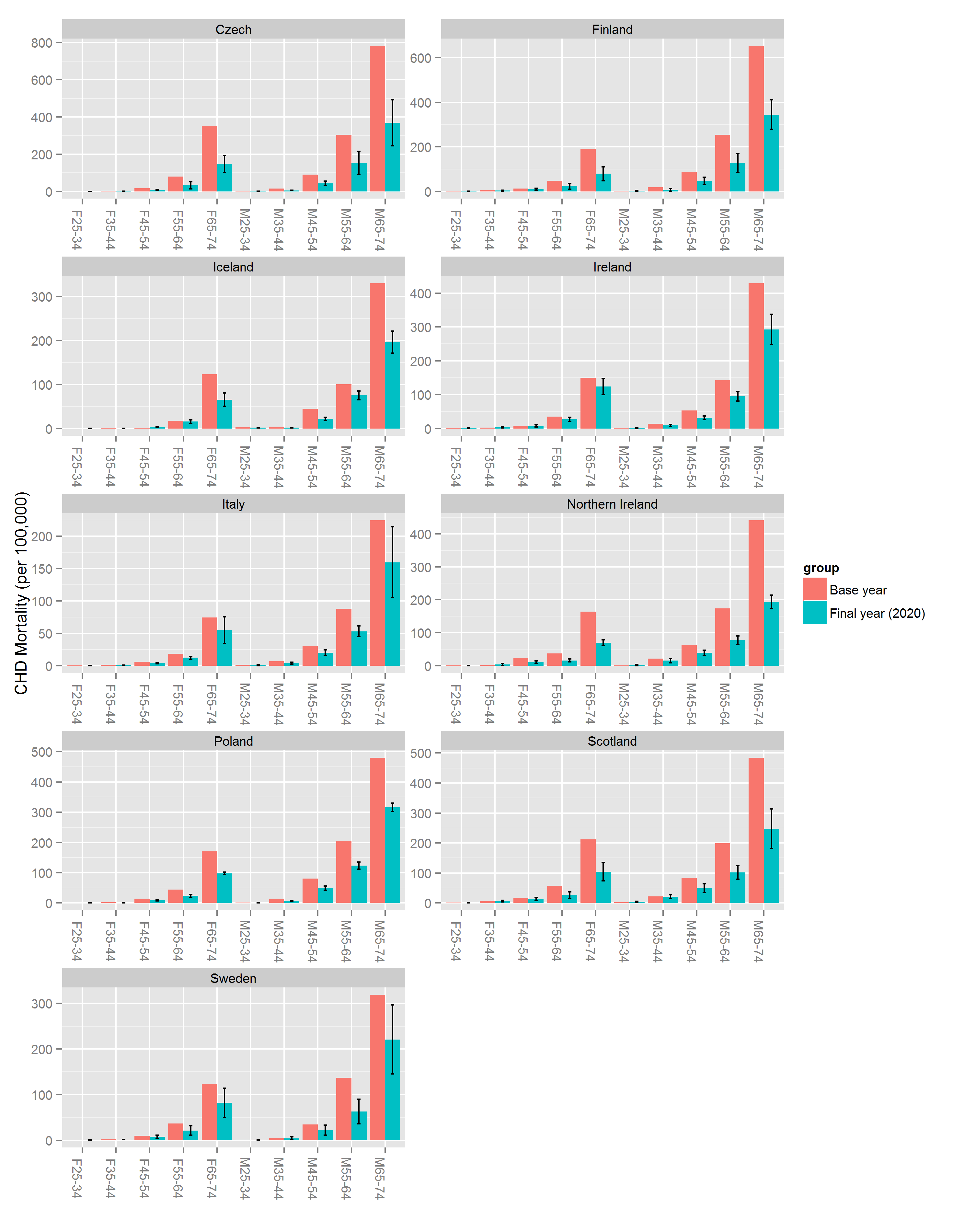


## A5. National Level Results

Table A5.1. Expected number of deaths prevented or postponed due to changes in salt consumption, according to three scenarios . S1 – conservative scenario, S2 – intermediate scenario, S3 – optimistic scenario.

| **Risk factor** | **Country** | **Forecast decrease in deaths [n]**  **(assumed no mortality change in future)** | | | **Forecast decrease in deaths [n]**  **(assumed current mortality trend according to exponential decay model)** | | | **Forecast decrease in deaths [%]**  **(assumed current mortality trend according to exponential decay model)** | | |
| --- | --- | --- | --- | --- | --- | --- | --- | --- | --- | --- |
|  |  | **S1** | **S2** | **S3** | **S1** | **S2** | **S3** | **S1** | **S2** | **S3** |
| Decrease in salt consumption | Czech Republic | 350 (160-570) | 650 (300-1080) | 920 (430-1530) | 160 (60-290) | 300 (120-560) | 420 (170-770) | 3.3 (1.3-6) | 6.1 (2.4-11.4) | 8.7 (3.4-15.8) |
|  | Finland | 100 (60-150) | 190 (120-280) | 280 (180-400) | 50 (30-80) | 100 (50-150) | 140 (80-220) | 2.3 (1.3-3.6) | 4.3 (2.4-6.8) | 6.2 (3.5-9.7) |
|  | Iceland | 3 (2-4) | 5 (4-8) | 8 (5-11) | 2 (1-3) | 3 (2-5) | 5 (3-7) | 2.7 (1.7-3.9) | 5.1 (3.2-7.4) | 7.2 (4.6-10.4) |
|  | Italy | 430 (270-620) | 800 (520-1170) | 1140 (740-1660) | 290 (160-460) | 550 (310-870) | 780 (440-1230) | 2.6 (1.5-4.2) | 5 (2.8-7.9) | 7.1 (4-11.2) |
|  | Northern Ireland | 20 (10-30) | 40 (20-50) | 50 (30-70) | 10 (10-10) | 20 (10-30) | 20 (10-40) | 2.1 (1.3-3.2) | 4 (2.4-6.1) | 5.7 (3.5-8.6) |
|  | Poland | 750 (480-1070) | 1400 (910-2020) | 1980 (1300-2840) | 450 (290-660) | 860 (550-1240) | 1210 (790-1750) | 3.3 (2.1-4.9) | 6.3 (4-9.1) | 8.9 (5.8-12.9) |
|  | Republic of Ireland | 50 (30-70) | 90 (60-140) | 130 (90-190) | 30 (20-50) | 70 (40-100) | 90 (60-140) | 2.3 (1.4-3.5) | 4.4 (2.7-6.6) | 6.3 (3.8-9.4) |
|  | Scotland | 70 (50-110) | 140 (90-200) | 200 (130-290) | 40 (20-60) | 70 (40-120) | 100 (60-170) | 2.2 (1.2-3.5) | 4.1 (2.3-6.5) | 5.9 (3.3-9.3) |
|  | Sweden | 120 (80-180) | 230 (150-330) | 330 (220-460) | 80 (40-130) | 150 (80-240) | 210 (110-330) | 3.2 (1.7-5.3) | 6.1 (3.2-9.9) | 8.6 (4.5-13.9) |
|  | **Total** | **1890 (1490-2330)** | **3550 (2840-4370)** | **5040 (4050-6180)** | **1120 (860-1410)** | **2100 (1640-2650)** | **2980 (2340-3770)** | **2.9 (2.3-3.7)** | **5.5 (4.3-7)** | **7.9 (6.2-9.9)** |

S1 – conservative scenario, S2 – intermediate scenario, S3 – optimistic scenario.

Table A5.2. Expected number of deaths prevented or postponed due to changes in proportion of consumed saturated/unsaturated fats, according to three scenarios . S1 – conservative scenario, S2 – intermediate scenario, S3 – optimistic scenario.

| **Risk factor** | **Country** | **Forecast decrease in deaths [n]**  **(assumed no mortality change in future)** | | | **Forecast decrease in deaths [n]**  **(assumed current mortality trend according to exponential decay model)** | | | **Forecast decrease in deaths [%]**  **(assumed current mortality trend according to exponential decay model)** | | |
| --- | --- | --- | --- | --- | --- | --- | --- | --- | --- | --- |
|  |  | **S1** | **S2** | **S3** | **S1** | **S2** | **S3** | **S1** | **S2** | **S3** |
| Decrease consumed saturated/unsaturated fats ratio | Czech Republic | 250 (120-400) | 480 (230-750) | 680 (330-1060) | 120 (50-210) | 220 (90-390) | 310 (130-550) | 2.4 (1-4.2) | 4.5 (1.8-8) | 6.4 (2.6-11.3) |
|  | Finland | 110 (80-140) | 200 (150-260) | 290 (210-370) | 50 (30-80) | 100 (60-150) | 150 (90-220) | 2.4 (1.4-3.6) | 4.6 (2.7-6.8) | 6.5 (3.9-9.6) |
|  | Iceland | 3 (2-3) | 5 (4-6) | 7 (6-8) | 2 (1-2) | 3 (2-4) | 4 (3-5) | 2.5 (1.9-3.2) | 4.7 (3.5-6) | 6.6 (5-8.4) |
|  | Italy | 420 (300-540) | 780 (570-1020) | 1100 (810-1440) | 270 (170-390) | 520 (330-740) | 730 (460-1050) | 2.5 (1.6-3.6) | 4.7 (3-6.8) | 6.6 (4.2-9.5) |
|  | Northern Ireland | 20 (20-30) | 40 (30-60) | 60 (50-80) | 10 (10-20) | 20 (10-30) | 30 (20-40) | 2.8 (1.8-4) | 5.3 (3.5-7.5) | 7.5 (4.9-10.5) |
|  | Poland | 570 (410-740) | 1070 (780-1400) | 1500 (1110-1970) | 340 (240-450) | 640 (460-850) | 900 (650-1210) | 2.5 (1.8-3.3) | 4.7 (3.4-6.3) | 6.6 (4.8-8.9) |
|  | Republic of Ireland | 60 (40-80) | 110 (80-140) | 150 (110-200) | 40 (30-60) | 70 (50-100) | 100 (70-150) | 2.7 (1.8-3.7) | 5 (3.4-6.9) | 7 (4.7-9.8) |
|  | Scotland | 90 (60-120) | 170 (120-220) | 230 (170-310) | 50 (30-70) | 90 (60-130) | 130 (80-190) | 2.8 (1.7-4) | 5.2 (3.2-7.5) | 7.3 (4.6-10.6) |
|  | Sweden | 90 (70-120) | 170 (130-220) | 240 (180-310) | 60 (30-90) | 110 (60-160) | 150 (80-230) | 2.4 (1.3-3.6) | 4.4 (2.4-6.8) | 6.3 (3.4-9.6) |
|  | **Total** | **1610 (1370-1870)** | **3020 (2580-3520)** | **4260 (3650-4970)** | **950 (770-1140)** | **1780 (1460-2140)** | **2510 (2070-3020)** | **2.5 (2-3)** | **4.7 (3.9-5.6)** | **6.6 (5.5-8)** |

S1 – conservative scenario, S2 – intermediate scenario, S3 – optimistic scenario

Table A5.3. Expected number of deaths prevented or postponed due to changes smoking prevalence, according to three scenarios . S1 – conservative scenario, S2 – intermediate scenario, S3 – optimistic scenario.

| **Risk factor** | **Country** | **Forecast decrease in deaths [n]**  **(assumed no mortality change in future)** | | | **Forecast decrease in deaths [n]**  **(assumed current mortality trend according to exponential decay model)** | | | **Forecast decrease in deaths [%]**  **(assumed current mortality trend according to exponential decay model)** | | |
| --- | --- | --- | --- | --- | --- | --- | --- | --- | --- | --- |
|  |  | **S1** | **S2** | **S3** | **S1** | **S2** | **S3** | **S1** | **S2** | **S3** |
| Decrease smoking prevalence | Czech Republic | 390 (120-700) | 780 (230-1410) | 1050 (300-1950) | 180 (50-350) | 360 (90-700) | 480 (120-970) | 3.6 (1-7.1) | 7.3 (1.8-14.2) | 9.9 (2.4-19.9) |
|  | Finland | 170 (60-290) | 330 (110-560) | 420 (140-740) | 90 (30-150) | 170 (50-300) | 220 (60-400) | 3.8 (1.2-6.9) | 7.4 (2.3-13.5) | 9.6 (2.9-17.9) |
|  | Iceland | 4 (1-6) | 8 (3-12) | 12 (4-19) | 2 (1-4) | 5 (2-8) | 8 (3-12) | 3.7 (1.4-5.8) | 7.6 (2.8-12.2) | 11.6 (4.1-19.1) |
|  | Italy | 610 (240-990) | 1240 (450-2010) | 1650 (600-2770) | 420 (150-720) | 840 (280-1460) | 1100 (370-1970) | 3.8 (1.3-6.6) | 7.6 (2.6-13.2) | 10 (3.4-18) |
|  | Northern Ireland | 30 (10-50) | 70 (30-110) | 100 (40-160) | 20 (10-20) | 30 (10-50) | 50 (20-80) | 3.8 (1.4-6) | 7.7 (2.8-12.5) | 11.5 (4.1-19.1) |
|  | Poland | 760 (240-1340) | 1430 (440-2600) | 1990 (600-3690) | 460 (140-810) | 870 (260-1560) | 1210 (360-2230) | 3.4 (1.1-5.9) | 6.4 (1.9-11.5) | 8.9 (2.7-16.4) |
|  | Republic of Ireland | 80 (30-120) | 160 (60-250) | 240 (90-390) | 50 (20-90) | 110 (40-180) | 170 (60-280) | 3.7 (1.4-5.9) | 7.4 (2.7-12.2) | 11.3 (3.9-18.8) |
|  | Scotland | 120 (50-190) | 250 (100-390) | 380 (140-600) | 70 (20-110) | 130 (50-220) | 200 (70-340) | 3.6 (1.4-6) | 7.4 (2.7-12.4) | 11.3 (3.9-19.1) |
|  | Sweden | 150 (60-250) | 310 (110-510) | 450 (150-750) | 90 (30-170) | 190 (60-360) | 280 (80-510) | 4 (1.2-7.2) | 8 (2.4-15) | 11.6 (3.2-21.5) |
|  | **Total** | **2330 (1520-3040)** | **4570 (2920-5960)** | **6300 (4000-8310)** | **1370 (860-1830)** | **2700 (1660-3600)** | **3710 (2270-5010)** | **3.6 (2.3-4.8)** | **7.1 (4.4-9.5)** | **9.8 (6-13.2)** |

Table A5.4. Expected number of deaths prevented or postponed in 2020 due to changes in prevalence of physical inactivity, according to three scenarios . S1 – conservative scenario, S2 – intermediate scenario, S3 – optimistic scenario.

| **Risk factor** | **Country** | **Forecast decrease in deaths [n]**  **(assumed no mortality change in future)** | | | **Forecast decrease in deaths [n]**  **(assumed current mortality trend according to exponential decay model)** | | | **Forecast decrease in deaths [%]**  **(assumed current mortality trend according to exponential decay model)** | | |
| --- | --- | --- | --- | --- | --- | --- | --- | --- | --- | --- |
|  |  | **S1** | **S2** | **S3** | **S1** | **S2** | **S3** | **S1** | **S2** | **S3** |
| Decrease physical inactivity prealence | Czech Republic | 170 (-50-500) | 340 (40-760) | 500 (80-1030) | 80 (-20-240) | 160 (20-370) | 230 (30-510) | 1.6 (-0.5-4.9) | 3.2 (0.3-7.6) | 4.7 (0.7-10.4) |
|  | Finland | 80 (-20-200) | 150 (20-300) | 220 (40-410) | 40 (-10-110) | 80 (10-160) | 110 (20-220) | 1.7 (-0.4-4.7) | 3.4 (0.5-7.2) | 5 (0.9-9.8) |
|  | Iceland | 1 (-1-4) | 3 (0-5) | 4 (1-7) | 1 (0-2) | 2 (0-3) | 3 (1-4) | 1.4 (-0.5-3.4) | 2.7 (0.4-5) | 4 (0.9-6.6) |
|  | Italy | 280 (-60-750) | 560 (90-1140) | 820 (170-1550) | 190 (-40-530) | 380 (60-820) | 560 (100-1120) | 1.8 (-0.4-4.8) | 3.4 (0.5-7.4) | 5.1 (0.9-10.2) |
|  | Northern Ireland | 10 (0-30) | 30 (0-50) | 40 (10-70) | 10 (0-20) | 10 (0-20) | 20 (0-30) | 1.5 (-0.5-4) | 3 (0.4-5.9) | 4.4 (0.9-7.9) |
|  | Poland | 370 (-1160-1910) | 720 (-700-2290) | 1060 (-290-2690) | 230 (-700-1190) | 440 (-420-1420) | 650 (-170-1670) | 1.7 (-5.1-8.8) | 3.2 (-3.1-10.4) | 4.8 (-1.2-12.3) |
|  | Republic of Ireland | 40 (-10-100) | 80 (10-160) | 110 (20-220) | 30 (0-70) | 50 (10-110) | 80 (10-160) | 1.8 (-0.2-4.9) | 3.6 (0.6-7.7) | 5.3 (0.9-10.5) |
|  | Scotland | 40 (-10-100) | 80 (10-150) | 120 (30-200) | 20 (-10-60) | 50 (10-80) | 70 (20-110) | 1.3 (-0.3-3.1) | 2.5 (0.4-4.7) | 3.7 (0.9-6.3) |
|  | Sweden | 70 (0-190) | 150 (20-300) | 200 (30-400) | 50 (0-130) | 90 (10-210) | 120 (20-270) | 2 (0-5.3) | 3.8 (0.6-8.7) | 5.2 (0.8-11.4) |
|  | **Total** | **1080 (-580-2670)** | **2100 (470-3760)** | **3080 (1340-4860)** | **640 (-360-1640)** | **1260 (260-2310)** | **1840 (770-2980)** | **1.7 (-0.9-4.3)** | **3.3 (0.7-6.1)** | **4.8 (2-7.9)** |

S1 – conservative scenario, S2 – intermediate scenario, S3 – optimistic scenario

**References**

[1] Lewington S, Clarke R, Qizilbash N, Peto R, Collins R, Age-specific relevance of usual blood pressure to vascular mortality: a meta-analysis of individual data for one million adults in 61 prospective studies. Lancet. 2002 Dec 14;360(9349):1903-13.

[2] Lewington S, Whitlock G, Clarke R, Sherliker P, Emberson J, Halsey J, Qizilbash N, Peto R, Collins R. Blood cholesterol and vascular mortality by age, sex, and blood pressure: a meta-analysis of individual data from 61 prospective studies with 55,000 vascular deaths. Lancet. 2008 Jul 26;372(9635):292.

[3] Ezzati M, Henley SJ, Thun MJ, Lopez AD. Role of smoking in global and regional cardiovascular mortality. Circulation. 2005 Jul 26;112(4):489-97.

[4] Bull F, Armstrong TP, Dixon T, Ham S, Neiman A, et al. (2004) Physical inactivity. In: Ezatti M, Lopez AD, Rodgers A, Murray CJL, editors. Comparative quantification of risk. Global and regional burden of disease attributable to selected major risk factors. Volume 1 ed. Geneva: World Health Organization. pp. 729-881.

[5] Joubert J, Norman R, Lambert EV, Groenewald P, Schneider M, et al. (2007) Estimating the burden of disease attributable to physical inactivity in South Africa in 2000. S Afr Med J 97:725-731.
